# A Prospective, Comparative Study to Evaluate the Diagnostic Accuracy of Mallampati Grading in Supine and Sitting Positions for Prediction of Difficult Airway

**DOI:** 10.7759/cureus.18465

**Published:** 2021-10-03

**Authors:** Abhishek Chatterjee, Vaibhav S Maheshwari, Pratap Rudra Mahanty, Deb Sanjay Nag, Rajiv Shukla

**Affiliations:** 1 Anaesthesiology, Tata Main Hospital, Jamshedpur, IND; 2 Anaesthesiology, Netaji Subhash Chandra Bose Medical College, Jabalpur, IND

**Keywords:** diagnostic accuracy of mallampati, mallampati grading in supine, mallampati grading in sitting, difficult intubation, difficult airway

## Abstract

Background & aim

Difficult airway is a major concern for all anaesthesiologists because failure to secure airway could lead to devastating complications or may increase morbidity and mortality. Airway assessment, therefore, is of paramount importance and anticipating a difficult airway prior to anesthetic administration, could help us in better preparation as well as avoidance of life-threatening complications. There are various tests available to assess the airway, out of which, modified Mallampati test (MLPT) is one of the common, easy and reliable methods to predict difficult airway. Mallampati test, usually is done with patient in sitting position. However, in certain group of patients in whom sitting position is not possible (suspected cervical spine injury, pelvic injury, patients in shock, etc.), the Mallampati test can be done in supine position. Few studies were available which concluded that Mallampati test in supine position was not only reliable but also superior to sitting position, whereas, few other studies contradicted this opinion. We, therefore, wanted to address this issue and tried to find out whether Mallampati test in supine position could offer better diagnostic accuracy or not.

Materials & methods

Mallampati test (MLPT) in sitting position was done in 100 patients initially in preoperative period and subsequently in supine position inside operating room prior to induction of anesthesia. During laryngoscopy, Cormack-Lehane (CL) grading was noted in all patients. Correlation of Mallampati test in sitting and supine position with Cormack-Lehane grading was obtained. A receiver operating characteristics (ROC) analysis was done to determine the area under the curve, the sensitivity and the specificity. Positive predictive value (PPV) and negative predictive value (NPV) were also calculated to analyse the diagnostic accuracy of Mallampati score (MLPT) in sitting and supine position.

Results

A toal of 22.2% of patients had difficult intubation (CL grade 3) although MLPT of these patients in sitting position anticipated a non-difficult airway (MLPT 1 and 2) and there was no significant correlation between MLPT grade in sitting position with the Cormack-Lehane grade. In comparison to sitting position, MLPT in supine position had significant correlation with the Cormack-Lehane grading and all patients with supine MLPT 1 and 2 (non-difficult airway) had easy intubation (CL grade 1 and 2). ROC analysis also showed that MLPT grade in supine position had superior correlation and better diagnostic accuracy than MLPT in sitting position for assessment of airway as indicated by higher sensitivity and better positive as well as negative predictive values.

Conclusion

Mallampati test done in supine position has far greater sensitivity and is superior in predicting difficult intubation as compared to MLPT done in conventional sitting position.

## Introduction

Difficult airway management has been a real challenge for anaesthesiologists, as failure to secure airway remains an important cause of hypoxic brain damage and other associated complications [1]. Detailed airway history along with clinical examination of airway is, therefore, of paramount importance during pre-anaesthetic check-up, so that difficult airway-related complications could be avoided.

The American Society of Anesthesiologists (ASA) defines the difficult airway as the clinical situation in which “a conventionally trained anaesthesiologist experiences difficulty with intubation, mask ventilation, or both” [1,2]. The Mallampati test (MLPT) is the most commonly performed test for the assessment of airway. Mallampati test depends on the visual inspection of oropharyngeal structures seen in patients in the sitting position with the head in neutral position, mouth open as widely as possible, and the tongue extended to its maximum without phonation [3,4]. However, the physical examination cannot always be performed in the sitting position, for example, in case of neck injury, spine injuries and pelvic injuries, where pain may limit mobilization. In view of such concerns, Hanouz et al. [5] conducted a study to find out the efficacy of Mallampati test in supine position and concluded that Mallampati test in supine position was superior to sitting position. In a similar study done by Bindra et al. [6], Mallampati test in supine position had a superior correlation with laryngoscopic view. However, contrary to both these studies, Tham et al. [4] observed that Mallampati test in supine position did not offer any advantages over Mallampati test in sitting position.

We, therefore, planned to conduct the present study to address the existing controversy regarding the superiority of Mallampati test in supine over sitting position.

## Materials and methods

This study was conducted in 940 bedded multi-disciplinary teaching hospital. Approval from the institutional ethics committee of Tata Main Hospital, Jamshedpur (where the study was conducted) was taken (approval no - 201-26104-182-219958, dated 27.11.2018 ) and written informed consent of all patients was obtained.

The sensitivity of Mallampati test in supine and sitting positions found in identical studies [5] ranges from 44% to 72%. Therefore, assuming sensitivity of 50% for Mallampati test and with 10% margin of error, the minimum required sample size at 5% level of significance was 96 patients. We therefore included 100 patients in our study.

Statistical analysis was done with the statistical package for the social science system (SPSS) version 17.0 (SPSS Inc., Chicago, IL). Continuous variables are presented as mean ± standard deviation and categorical variables are presented as absolute numbers and percentage. Nominal categorical data between the groups were compared using Chi-square test or Fisher’s exact test as appropriate. A receiver operating characteristics (ROC) analysis was done to determine the area under the curve, the sensitivity and the specificity. Positive predictive value and negative predictive value were also calculated to analyse the diagnostic accuracy of Mallampati score (MLPT) in sitting and supine position correlating with Cormack-Lehane (CL) grade. For all statistical tests, a p-value less than 0.05 was taken to indicate a significant difference.

Patients of 17-50 years of age group belonging to ASA1 and ASA2 grade scheduled for orthopaedic, urologic, gynaecologic, and abdominal surgery were included and it was ensured that airway evaluation would be possible both in sitting as well as supine position and patients were in need of oro-tracheal intubation for surgeries.

Pregnant patients or patients belonging to ASA3 and ASA4 or posted for head, neck, thoracic surgeries or with limited mouth opening (less than 3 cm) were excluded.

**Figure 1:**
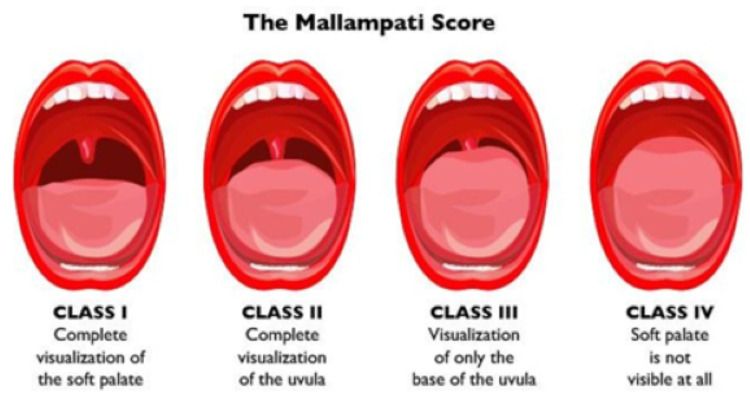
Mallampati test Source: clinicaladvisor.com

During the pre-anaesthesia consultation, the airway examinations included Mallampati test observed in the sitting position with the head in neutral position, mouth fully open, tongue out, and without phonation (Figure 1) [5].

Before induction of anesthesia, the Mallampati test was evaluated in supine position on the operating room table (using a 5-cm-high gel donut head pillow and neck in neutral position, mouth fully open, tongue out, and the observer placed at the patient’s head looking at oropharyngeal structures from above, with eyes aligned on a vertical axis starting from the middle of the opened mouth) [5,7]. The observer in the operation theatre was the same individual who had observed the patient preoperatively and noted MLPT in sitting position.

After shifting to the operation theatre, all monitors as per ASA minimum monitoring standards were attached. Following pre-oxygenation, all patients received inj. midazolam 1 mg, inj. fentanyl 2 mcg/kg body weight and induced with inj. propofol at a dose 2 mg/kg body weight. After checking for ventilation, inj. vecuronium at 0.1 mg/kg body weight was administered and endotracheal intubation was done after three minutes.

**Figure 2:**
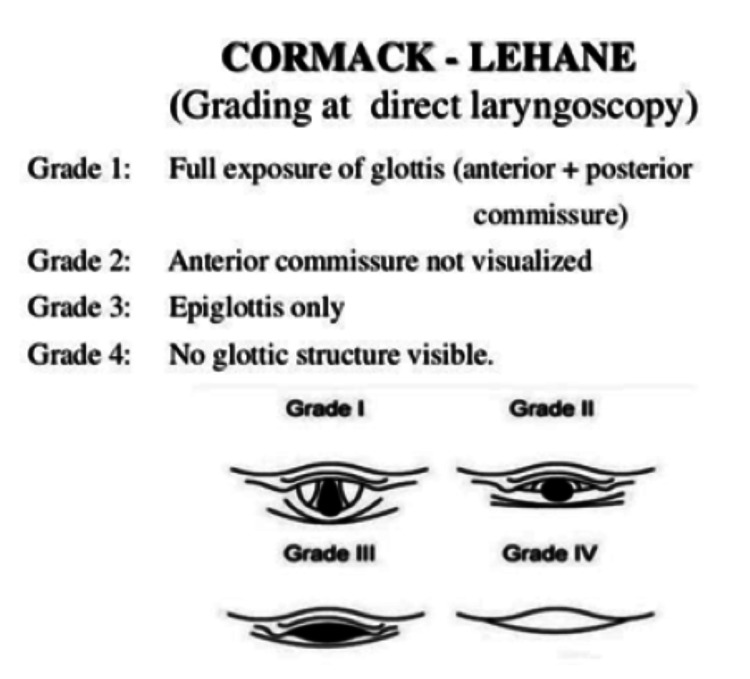
Cormack-Lehane grading during laryngoscopy

Cormack-Lehane Grade (CL) scores I and II were classified as easy intubations whereas grades III and IV were classified as difficult intubations (Figure 2).

## Results

The sample size of our study, as described above was 100. The mean age of the sample population was 36.58 ± 8.40 years. Fifty-five percent of the patients were females while 45% were males. It was observed that 46% of the patients were in the BMI range of 18.5-24.9 and 54% were in the range of 25-29.9. Twenty-eight percent of the patients belonged to ASA grade 1, whereas 72% belonged to ASA grade 2. Endotracheal intubation was found to be easy in 82 patients, difficult in 17 patients and one patient needed awake fibreoptic intubation. We, therefore, included 99 patients for analysis of our data.

For MLPT (sitting) grade 1, only 30.8% had MLPT grade 1 in supine position, whereas 61.5% of the patients had MLPT grade 2 and 7.7% became MLPT grade 3 in supine position. For MLPT (sitting) grade 2, 50% of the patients remained MLPT grade 2 whereas 48.4% migrated to MLPT grade 3 in supine position. Similarly, for MLPT (sitting) grade 3, 58.3% of the patients remained MLPT grade 3, whereas 25% had MLPT grade 4 and 16.7% had a better grade 2 in supine position. However, for the MLPT (sitting) grade 4, 100% of the patients had the same MLPT grade of 4 in supine position. The changes in MLPT grade from sitting to supine position are statistically significant (Table 1).

**Table 1:** Co-relation between Mallampati (MLPT) grade sitting and supine positions

MLPT grade (sitting)	Total	MLPT grade (supine)				P-value
		1	2	3	4	
		Frequency (%)	Frequency (%)	Frequency (%)	Frequency (%)	
1	13	4 (30.8%)	8 (61.5%)	1 (7.7%)	0 (0.0%)	<0.001
2	62	1 (1.6%)	31 (50.0%)	30 (48.4%)	0 (0.0%)	
3	24	0 (0.0%)	4 (16.7%)	14 (58.3%)	6 (25.0%)	
4	1	0 (0.0%)	0 (0.0%)	0 (0.0%)	1 (100%)	
Total	100	5 (5.0%)	43 (43.0%)	45 (45.0%)	7 (7.0%)	

A total of 7.7% of the patients had Cormack-Lehane (CL) grade 3 (difficult intubation) who had MLPT grade 1 (non- difficult airway) in sitting position. Similarly, 14.5% of the patients had CL grade of 3 (difficult intubation) in whom the MLPT score was 2 (non-difficult airway) in sitting position. In this way, it was observed that 22.2% of patients had difficult intubation (CL grade 3) although MLPT of these patients in sitting position anticipated a non-difficult airway (MLPT 1 and 2). For the MLPT grade 3 (difficult airway) in sitting position, 25% of the patients had Cormack-Lehane grade of 1 (easy intubation), while 45.8% of the patients had Cormack-Lehane grade of 2 (easy intubation). It was, therefore, observed that there was no significant correlation between MLPT grade in sitting position with the Cormack-Lehane grade with p-value of 0.090 (Table 2).

**Table 2:** Correlation between Mallampati grade sitting and Cormack-Lehane (CL) grades

MLPT grade (sitting)	Total	Cormack-Lehane (CL) grade			P-value
		1	2	3	
		Frequency (%)	Frequency (%)	Frequency (%)	
1	13	11 (84.6%)	1 (7.7%)	1 (7.7%)	0.090
2	62	36 (58.1%)	17 (27.4%)	9 (14.5%)	
3	24	6 (25.0%)	11 (45.8%)	7 (29.2%)	

For the supine MLPT grade 1 (non-difficult airway), 100% of the patients had Cormack-Lehane grade of 1 (easy intubation). For the MLPT grade 2 (non-difficult airway) in supine position, 79.1% of the patients had Cormack-Lehane grade of 1 and 20.9% of the patients had Cormack-Lehane grade of 2, i.e., 100% patients had easy intubation (Cormack-Lehane grade 1 and grade 2 are considered as easy intubation). For the MLPT grade 3 (difficult airway) in supine position, 31.1% of the patients had Cormack-Lehane grade of 1 while 42.2% had Cormack-Lehane grade of 2 and 26.7% had Cormack-Lehane grade of 3. It was, therefore, observed that there was a significant correlation between MLPT grade in supine with the Cormack-Lehane grading with a p-value of <0.001 (Table 3).

**Table 3:** Correlation of Mallampati grades supine with Cormack-Lehane (CL) grading

MLPT (supine)	Total	Cormack-Lehane (CL) grade			P-value
		1	2	3	
		Frequency (%)	Frequency (%)	Frequency (%)	
1	5	5 (100%)	0 (0.0%)	0 (0.0%)	<0.001
2	43	34 (79.1%)	9 (20.9%)	0 (0.0%)	
3	45	14 (31.1%)	19 (42.2%)	12 (26.7%)	
4	6	0 (0.0%)	1 (16.7%)	5 (83.3%)	

**Figure 3:**
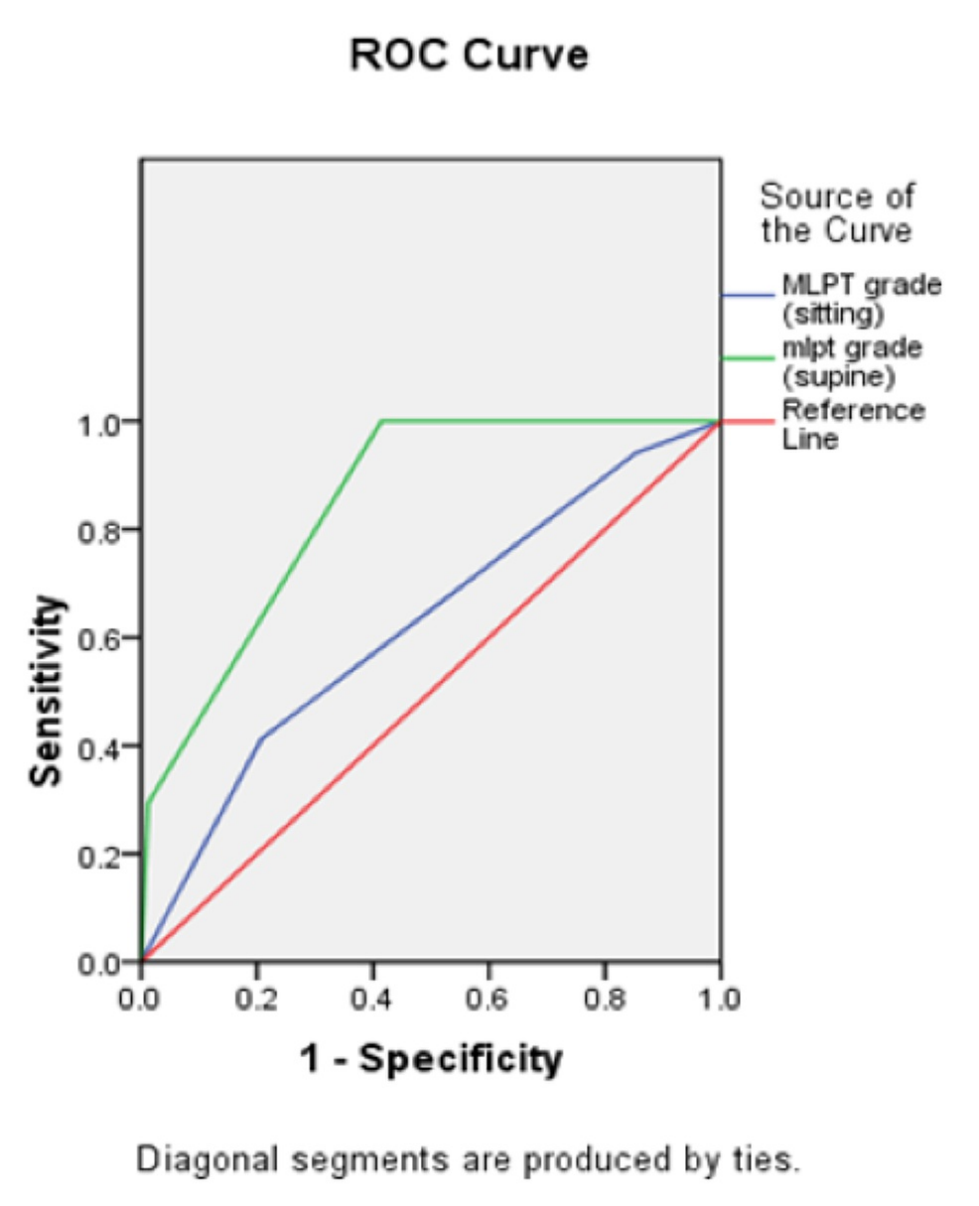
Receiver Operating Characteristics (ROC) curve

Receiver operating curve was used to predict the diagnostic accuracy of MLPT grade in sitting and supine position correlating with difficult Cormack-Lehane grade (Figure 3).

We observed that the area under the curve of MLPT grade in sitting position was 0.622 (95% CI 0.474-0.770). The sensitivity, specificity, positive predictive value (PPV) and negative predictive value (NPV) were 41.2%, 79.3%, 29.2% and 86.7% in predicting difficult Cormack-Lehane grade. However, the area under the curve for supine position was 0.848 (95% CI 0.765-0.930). The sensitivity, specificity, PPV and NPV was 100%, 58.5%, 33.3% and 100% for MLPT >=3 and 29.4%, 98.8%, 83.3%, 87.1% for MLPT grade >=4 in predicting difficult CL grade. In this way, it was observed from ROC analysis that MLPT grade in supine position has a superior correlation with better diagnostic accuracy than MLPT in sitting position for assessment of airway (Tables 4, 5).

**Table 4:** Area under ROC curve MLPT: Mallampati test

Test Result Variable(s)	Area	Std. Error	Asymptotic Sig.	Asymptotic 95% Confidence Interval	
				Lower Bound	Upper Bound
MLPT grade (sitting)	0.622	0.076	0.115	0.474	0.77
MLPT grade (supine)	0.848	0.042	0	0.765	0.93

**Table 5:** Sensitivity, specificity, positive predictive value, negative predictive value and accuracy of Mallampati in sitting and in supine position MLPT: Mallampati test; PPV: Positive predictive value; NPV: Negative predictive value.

Sitting MLPT Grade	Sensitivity	Specificity	PPV	NPV	Accuracy	P-value
>=3	41.20%	79.30%	29.17%	86.67%	72.73%	0.073
Supine MLPT Grade	Sensitivity	Specificity	PPV	NPV	Accuracy	p-value
>=3	100.00%	58.50%	33.33%	100.00%	65.66%	<0.001
>=4	29.40%	98.80%	83.33%	87.10%	86.87%	<0.001

## Discussion

The American Society of Anesthesiologists recommended that, whenever feasible, an airway history and physical examination should be conducted in all patients before the initiation of anaesthetic care, to avoid hypoxic injuries to the brain. To date, despite extensive advancement in airway management, failure to secure airway remains an important cause of hypoxic brain damage and other associated complications [1,2,8].

In order to avoid such disastrous consequences and rule out difficult airway, we perform several clinical tests, which includes the Mallampati test (MLPT), inter-incisor distance, sternomental and thyromental distances, relationship of maxillary and mandibular incisors, cervical spine mobility, and neck anatomy [7-13]. However, Mallampati test is still the most popular morphologic test used to evaluate the airway [14].

O’leary et al. [15], who reviewed the work of Mallampati et al. [7], stated that the original article did not report a positive predictive value, nor sensitivity or specificity, but it was calculated from the data and the positive predictive value was 14/15 (93.3%). But while reviewing other literatures, they found no studies that reported such high positive predictive value but observed a wide range of positive predictive values from 13% to 65% [15]. Tse et al. [16], in their study, reported that Mallampati class 3 test had a sensitivity of 66%, and a positive predictive value of 22%, whereas Lee et al. [14] in their meta-analysis reported MLPT had sensitivity of 0.55, specificity of 0.84, positive predictive value of 3.54 and negative predictive value of 0.54. Considering all these results of the above studies in favour of Mallampati test as done by various researchers across the globe, we, in the present study have therefore taken Mallampati test as the clinical test for assessment of difficult airway.

Contrary to the conventional way of Mallampati test being done in sitting position, Khan et al. hypothesised that the Mallampati test could be performed in the supine position without phonation with equally good results and thus conducted the study to find the result for so [17]. Hanouz et al. [5] in a similar study tried to find a different approach to assess the airway by Mallampati, when it is not viable to perform in every condition i.e. in an emergency scenario, where pain may limit mobilisation or where immobilisation of the spine is of utmost necessity in case of spinal injuries. So, in order to know the diagnostic performance, they conducted the study to detect the accuracy of Mallampati grade in sitting and supine position and observed that the Mallampati test in supine position was also effective in the assessment of difficult airway. However, studies undertaken by Tham et al. [4] and Amadasun et al. [18], who also did their study with similar methodology, did not find convincing results in favour of Mallampati done in supine position. We, therefore, in the present study tried to address this issue and hence performed Mallampati in sitting as well as in supine position for assessment of difficult airway.

In the present study, we examined MLPT in sitting position initially and then the patients were made supine and MLPT was done in supine position. In this way, we tried to find out whether MLPT grade changes from sitting to supine or not. While studying this change, we have found that patients with Mallampati grade 1 in sitting positions, when they were examined in supine position, only 30.8% remained grade 1 in supine, whereas 61.5% had grade 2 and 7.7% became grade 3 MLPT. On further analysis, we have observed that this 7.5% of MLPT 3 in supine (who had MLPT 1 in sitting) also had Cormack-Lehane 3 with difficult intubation (Tables 2, 3). This finding indicates that MLPT in supine position had better co-relation with difficult intubation.

Similarly, for the MLPT grade 2 in sitting, 50% of the patients remained MLPT grade 2 in supine position whereas, 48.4% became MLPT grade 3 and 1.6% became grade 1. For the MLPT grade 3 in sitting, 58.3% of the patients remained MLPT grade 3 in supine, whereas 25% became MLPT grade 4 and 16.7% had MLPT grade 2 in supine position. For the MLPT grade 4 in sitting, all patients remained 100% in supine position. Patients with Mallampati grade 4 in sitting remained in the same grade after assuming supine position, i.e. with the increase in Mallampati grade, the effect of the patient’s position became non-significant.

Singhal et al. [19] also observed similar results when patients were made supine from sitting position (P < 0.0001) and this change was always towards a higher grade when the patient was turned supine from the sitting position. Hanouz et al. [5] also showed a similar moderate relationship between the sitting and supine MLPTs.

In our study, when Mallampati grades in sitting position were compared with Cormack-Lehane grade (Table 2), one patient (7.7%) had a Cormack-Lehane of grade 3 (difficult intubation) who had Mallampati grade 1 (non-difficult airway) and out of 62 patients with grade 2 Mallampati grade (non-difficult airway), nine patients (14.5%) had Cormack-Lehane of grade 3 (difficult intubation). On finding the co-relation between the two, the p-value came out to be 0.09 which showed non-significant correlation between Cormack-Lehane grade and Mallampati test in sitting position. However, when Mallampati test in supine position was compared with Cormack-Lehane grading (Table 3), it showed a significant co-relation with a p-value of <0.001. None of the patients with Mallampati grade 1 (supine) had Cormack-Lehane grade 3 or 4. The same was also observed with Mallampati grade 2. For Mallampati grade 3 (difficult airway) in supine position, 42.2% had grade 2 Cormack-Lehane (CL) and 31.1% had grade 1 CL (easy intubation).

Ul Haq and Ullah [20], also tried to find out the correlation between MLPT in sitting with CL grading and had observed that patients who had anticipated easy intubation with Mallampati grade 1 (sitting position), 11.16% of them had Cormack-Lehane grade 3 (7.7% in our study) and among grade 2 Mallampati patients, 26.6% of patients had Cormack-Lehane 3 as compared to 14.5% in our study. On combining the results, 22.5% of patients of our study (as compared to 37.76% in the study by Ul Haq and Ullah) had difficult intubation (CL grade 3), although they had been classified as non-difficult airway (grade 1 and 2) when assessed by MLPT in sitting position.

Awasthi et al. [21] did a similar study in paediatric age group and found that those patients who were predicted to be easy as per Mallampati grade in sitting (without phonation), 2% of them had higher Cormack-Lehane grades. While comparing it in supine position only 1.3% of them had higher grades in Cormack-Lehane grade.

In the present study, we did not observe any significant correlation between MLPT grade sitting with the Cormack-Lehane grade (p-value 0.090). However, contrary to this result, we observed a significant correlation between MLPT grade supine with the Cormack-Lehane grade (p-value <0.001) indicating that Mallampati supine is a superior parameter in comparison to Mallampati sitting in the prediction of visualisation of vocal cords during laryngoscopy and endotracheal intubation.

The receiver operating curve (Figure 3), the area under the curve (Table 4) of Mallampati grade in sitting position came out to be 0.622 (95% CI 0.474-0.770) which was lower as compared to 0.848 (95% CI 0.765-0.930) in supine position. While comparing the sensitivity, specificity, positive predictive value and negative predictive value in both sitting and supine positions with Cormack-Lehane grading, the Mallampati sitting had lower sensitivity, positive predictive value and negative predictive value as compared to MLPT in supine position. Statistical analysis of ROC curve in the present study is consistent with the study undertaken by Hanouz et al. [5] which shows a similar finding of the ROC curve for the MLPT in supine position (0.82 [0.78-0.84]) and for the MLPT in the sitting position (0.70 [0.66-0.75]; P < .001).

Based on the above findings, we can, therefore, assume that the MLPT performed in the supine position is possibly superior in anticipating difficult airway as compared to MLPT performed in the sitting position.

## Conclusions

The results of the present study found out that a difficult airway was encountered in a significantly higher proportion of patients (22.2%) who had Mallampati grades 1 and 2 (non-difficult airway) in sitting position, whereas, none of the patients showed such aberrations in supine position. Besides this, the statistical analysis also showed that Mallampati grading in supine position not only had higher sensitivity, but also had superior positive as well as negative predictive values in comparison to sitting position. Further, it was observed that performing the Mallampati test in supine position is much more convenient for patients than in sitting position, especially in patients with injury to pelvis, hip or neck.

Based on the above findings, we, therefore, can conclude that Mallampati test, if performed in supine position, has a superior efficacy in diagnosing difficult airway when compared with Mallampati test in conventional sitting position.
